# A Case Report of Surgical Approach in Managing De Quervain’s Tenosynovitis

**DOI:** 10.7759/cureus.60373

**Published:** 2024-05-15

**Authors:** Ishiqua V Patil, Gajanan Pisulkar, Khizar K Khan, Ankur Salwan, Kevin Kawade

**Affiliations:** 1 Hospital Administration, Jawaharlal Nehru Medical College, Datta Meghe Institute of Higher Education and Research, Wardha, IND; 2 Orthopedic Surgery, Jawaharlal Nehru Medical College, Datta Meghe Institute of Higher Education and Research, Wardha, IND; 3 Orthopedics, Jawaharlal Nehru Medical College, Datta Meghe Institute of Higher Education and Research, Wardha, IND

**Keywords:** abductors pollicis longus, physical therapy rehabilitation, corticosteroid, nonsteroidal anti-inflammatory medication, tenosynovitis

## Abstract

De Quervain's tenosynovitis is characterized by swelling of muscles (abductor pollicis longus (APL) and extensor pollicis (EPB) brevis), and they are located at the base of the thumb. This is a very irritating and painful condition. In many cases, late detection causes an increase in inflammation, and due to prolonged ignorance and neglect, the patient suffers from pain and discomfort that affects and restricts their daily routine work. The disorder tenosynovitis is triggered by preexisting tendon degeneration induced by excessive twisting actions. Inflammatory arthritis is primarily associated with the disorder. The tendon sheath thickens and becomes constricted if the inflammation and swelling persist. Patients who undergo high-torque wrist turning or other repetitive everyday movements, such as handshaking, have a higher risk of developing tenosynovitis. This disease can also occur without any sort of visible prior trauma or injury. Clinical evaluation is usually required for diagnosis; however, imaging studies might be used to confirm the diagnosis or check out alternate diseases. Nonsteroidal anti-inflammatory medication (NSAIDs), physical therapy, immobilization with splints, and rest are among the treatment options. Applying ice to the affected area and applying a splint are a few ways to ease the pain. Corticosteroid injections or surgery may be considered in situations that do not respond to preventive treatment; thus, patients are advised to go for minor surgery to get relief from prolonged pain.

## Introduction

Fritz De Quervain originally proposed the theory of stenosing tenosynovitis within the radial dorsum of the wrist. Radial styloid tenosynovitis, commonly known as de Quervain's tenosynovitis, is a disorder that affects the tendon on the thumb side of the wrist. The tendon that allows the thumb's action is irritated and swollen in this condition [1]. The tendons involved are the extensor pollicis brevis (EPB) and abductor pollicis longus (APL) enclosed in a common sheath at the dorsum of the wrist. The thumb and wrist experience swelling, tenderness, pain, and discomfort due to this sheath inflammation [2]. Adults from 30 to 60 years of age and those who use their hands and wrists more frequently are prone to this condition. This disorder can develop from activities such as holding, lifting, or twisting the wrist [3]. This medical condition is more frequently seen among those who work in occupations or have hobbies that require repeated hand motion. Over time, the condition manifests as growing pain, swelling, stiffness, and sensitivity in the hand at the wrist, below the base of the thumb [4]. This pain usually worsens with thumb and wrist movements and can radiate a feeling up the forearm.

In severe cases, affected individuals may experience difficulty in performing day-to-day tasks that involve thumb and wrist usage [5]. Despite its impact on daily activities, de Quervain's tenosynovitis is generally a condition that can be effectively managed with appropriate treatment and, if necessary surgery [6]. Diagnosing radial styloid tenosynovitis involves a clinical examination by a healthcare professional, notably the Finkelstein test, where the patient makes a fist with the thumb tucked within the fingers and then bends the wrist toward the little finger, a commonly used diagnostic technique [7]. Initial treatment typically involves essential medication for pain relief. When these measures fail to provide relief, surgery may be considered. The primary goal of surgical treatment is to release the constricted tendon sheath, allowing the tendons to move freely. This procedure, known as tendon sheath release, is often performed under local anesthesia. It involves a small incision over the radial side of the wrist, through which the constricting sheath is cut to alleviate pressure on the tendons. Post-operative care includes a combination of medications, hand therapy, and a gradual return to regular activities [8].

## Case presentation

A 56-year-old male patient was referred to Acharya Vinoba Bhave Rural Hospital Wardha. The patient came with a history of restricted movement of the left hand's wrist especially in the thumb area, for the past six months. In the prior history of the patient, he expressed that he tried cold compressions and pain killers but they worked temporarily; once stopped consuming the pain returned back, and at present it was hurting so badly that he was not able to do his daily work. On physical examination, he had a pulse rate of 86 bpm, BP of 120/80, and saturation of 98% at room air. The patient showed swelling and soreness over the tendon, which was unbearable as he was working and using his hand despite the pain. The patient was examined with Finkelstein's test to check for tenosynovitis in the wrist's EPB and APL tendons; he was reported positive for the test. With hand movement, there was a creaking sound, thumb flexion and extension, and a catching or stop-and-go sensation. On observation, based on the scan, and ultrasound report, it was seen that the patient's tendon was inflamed badly; despite trying all non-surgical treatments, his condition had no changes instead his pain gradually increased and was getting unbearable; thus, he was suggested to undergo a surgical procedure for permanent pain relief. In many cases, joint immobilization, rest, and an ice pack decrease the discomfort. In rare circumstances, physiotherapy and corticosteroid injections are used as treatments; surgery is advised if non-surgical treatments fail. Thus, the patient was counseled and educated about the surgical procedure under local anesthesia. A skin incision was made on the wrist side, adjacent to the base of the thumb, to relieve the tight band covering the swollen parts of the tendon during the surgical release, which allows the tendon to move freely without pain. Doctors also suggest wearing a splint on the operated hand for one to four weeks after surgery and to heal completely; the healing requires six to 12 weeks. Patients' diet, medication, and follow-up were scheduled properly at the intervals of the month for speedy and healthy recovery.

Clinical examination

On assessment, the patient's height was 177 cm, and a weight of 59 kg was observed, along with a pulse rate of 86 bpm and blood pressure of 120/80 per minute. Examination of the right hand revealed swelling and severe deformation. Thumb abduction is uncomfortable. Finkelstein's test was positive. The distal circulation and movement are intact. The patient has diabetes mellitus for three months and is now taking medication. The patient had no history of hypertension, pulmonary tuberculosis, bronchial asthma, or COVID-19, and he did not go through any severe surgery before.

Treatment

**Figure 1:**
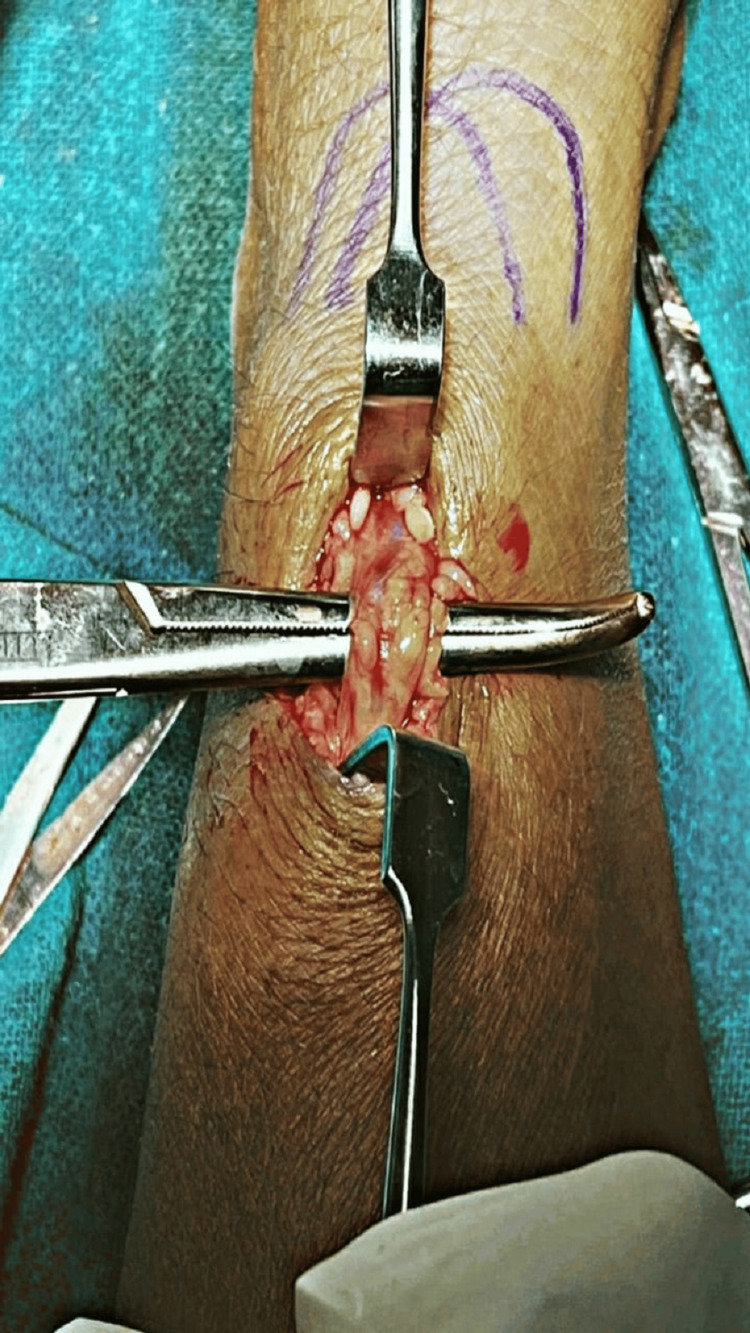
APL and EPB APL, abductor pollicis longus; EPB, extensor pollicis brevis

On the basis of investigations, clinical examinations, and tests done, the patient was diagnosed with De Quervain's disease (DQD) as he tested positive for Finkelstein's test, and when non-surgical treatment did not work, the patient was suggested to undergo minor surgery for the release of muscle to ease the pain. The surgery was performed to release the tendon sheath for De Quervain's tenosynovitis of the right hand in the wrist. The operation took place under local anesthesia, and the overall procedure took 30 minutes. The local anesthesia was injected into the tendon sheath around the affected tendon. 4 mL per site of injection was given to the patient. A 2 cm incision was made over the left anatomical snuff box. Proper precautions were taken during the operation to avoid infection rates. Following the incision, the skin and soft tissue were meticulously incised and retracted to expose the underlying structures. During the procedure, the radial nerve was identified and pulled back dorsally. The closure of the incision was carried out using Vicryl 1-0 and Ethilon 2-0 sutures. The medication prescribed was an antibiotic regimen, including cefuroxime 500 mg twice daily for five days. For pain management, the patient was advised to take Zerodol SP twice daily, providing analgesic relief to alleviate post-operative discomfort. Pantop 40 mg was prescribed once daily before consuming food. These prescribed medications were given to optimize the patient's recovery, mitigate pain, and reduce the risk of complications. Figure 1 shows the tendon sheath of the EPB and abductor pollicus longus (APL), and they were cut to release the tension.

Follow-up

The patient was satisfied and responded positively to the surgical procedure and medication given for the recovery. He recovered within a period of four to six weeks and was able to perform daily routine tasks generally without pain.

## Discussion

DQD is often referred to as a painful disorder involving the wrist's lateral tendons. It is the inflammation of the tendons (the EPB and APL) that are responsible for thumb movement. These tendons run through a tiny tunnel called the wrist's first extensor compartment. Considering such conditions, the fibro-osseous sheath tightens and becomes inflamed. It affects adults, primarily women aged 30 to 50 years, especially those whose daily jobs include continuous hand or wrist motions [9]. Yet, it can occur to men and women of any age who engage in repetitive hand tool usage or sports-related activities that strain the tendons in their hands and wrists. This was initially discovered by Fritz De Quervain in 1895. He hypothesized that the illness was caused by repeated strain among individuals who are involved in wrist-intensive activities (such as assembly workers) [10]. The illness usually produces discomfort and soreness across the forearm and on the lateral side of the wrist. Along with inflammation, patients may experience difficulty gripping objects or moving their thumbs. Repetitive hand and wrist motions, like pinching, twisting, and grabbing, might make the condition severe [11]. The preliminary research implies that DQD is not carried on by an underlying inflammatory illness but rather by myxoid degeneration [12]. Several risk factors, like exposure to somatotropin and hereditary susceptibility, have also been reported [13]. Numerous studies have found anatomical changes in the significant dorsal extensor compartment, which has been demonstrated to affect the effectiveness of therapy [14].

Therefore, it has been reported that anatomical fluctuation leads to various success rates with different therapeutic regimens. Considering the particular wrist architecture of each patient, changes may be required to perform physical therapy, corticosteroid injections, and therapeutic ultrasonography [15]. Evaluating the effectiveness of various medications in huge population groups has provided insights into the underlying causes and the constantly changing treatment method for DQD [16].

## Conclusions

People with de Quervain's tenosynovitis face a great deal of difficulty because of the severe symptoms they often experience, such as discomfort, swelling, and restricted thumb and wrist movement. The condition can seriously affect everyday work, with gradually increasing pain in the wrist and sometimes extending to the arms. Although awareness of the condition, as well as its diagnosis and treatment decisions, is necessary for recovery, sometimes non-surgical procedures benefit the patient, and sometimes the condition has to be treated by a minor operation that helps the patient to recover within three to six weeks with some oral medication and a splint suggested by the physician for better mobilization and recovery.
